# Supplementary material for: Discrete or indiscrete? Redefining the colour polymorphism of the land snail Cepaea nemoralis
Source: Heredity (Edinb). 2019 Feb 26;123(2):162–75. doi: 10.1038/s41437-019-0189-z (PMC6629550; doi:10.1038/s41437-019-0189-z)
Supplement: Supplementary file 1 — Supplementary material info [file 41437_2019_189_MOESM1_ESM.pdf]

Supplementary Fig. 1. Boxplot showing extent of achromatic variation in Mclust-defined colour morphs of *Cepaea nemoralis*.

Supplementary Fig. 2. Scatterplot and associated density plot, showing variation of visual space coordinates, xyz, along three principal component axes. The six clusters that Mclust recovered are shown as coloured circles. Yellow circles loosely correspond to a subset of human-perceived yellow and pink shells but otherwise the concordance is poor. Top: PC1 versus PC2. Bottom: PC2 versus PC3.

Supplementary Movie 1. Animation showing axes of chromatic variation in the shell of *C. nemoralis*, using avian visual space. Units on x, y and z axes are in JNDs. The solid lines illustrate variation along the first three principal components; individual points are coloured according to human-scoring of the shell, either yellow, pink or brown.

Supplementary Movie 2. Animation showing axes of chromatic variation in the shell of *C. nemoralis*, using avian visual space. Units on x, y and z axes are in JNDs. The solid lines illustrate variation along the first three principal components; individual points are coloured according to Mclust classification of the shell, either yellow, pink or brown.

Supplementary Movie 3. Animation showing axes of chromatic variation in the shell of *C. nemoralis* from the Beeston population sample, using avian visual space. Units on x, y and z axes are in JNDs. Individual points are coloured according to human-classification of the shell, either yellow or pink.

Supplementary Movie 4. Animation showing axes of chromatic variation in the shell of *C. nemoralis* from the Beeston population sample, using avian visual space. Units

on x, y and z axes are in JNDs. Individual points are coloured according to Mclust-classification of the shell, either yellow, pink or brown.

Supplementary Movie 5. Animation showing axes of chromatic variation in the shell of *C. nemoralis* from the Bathhill population sample, using avian visual space. Units on x, y and z axes are in JNDs. Individual points are coloured according to human-classification of the shell, either yellow, pink or brown.

Supplementary Movie 6. Animation showing axes of chromatic variation in the shell of *C. nemoralis* from the Bathhill population sample, using avian visual space. Units on x, y and z axes are in JNDs. Individual points are coloured according to Mclust-classification of the shell, either yellow, pink or brown.

Table S1. Source of snails used in this study.

Table S2. Qualitative phenotype and quantitative reflectance data for the samples used in this study.
