# Supplementary figures and images for: Discrete or indiscrete? Redefining the colour polymorphism of the land snail Cepaea nemoralis
Source: Heredity (Edinb). 2019 Feb 26;123(2):162–75. doi: 10.1038/s41437-019-0189-z (PMC6629550; doi:10.1038/s41437-019-0189-z)

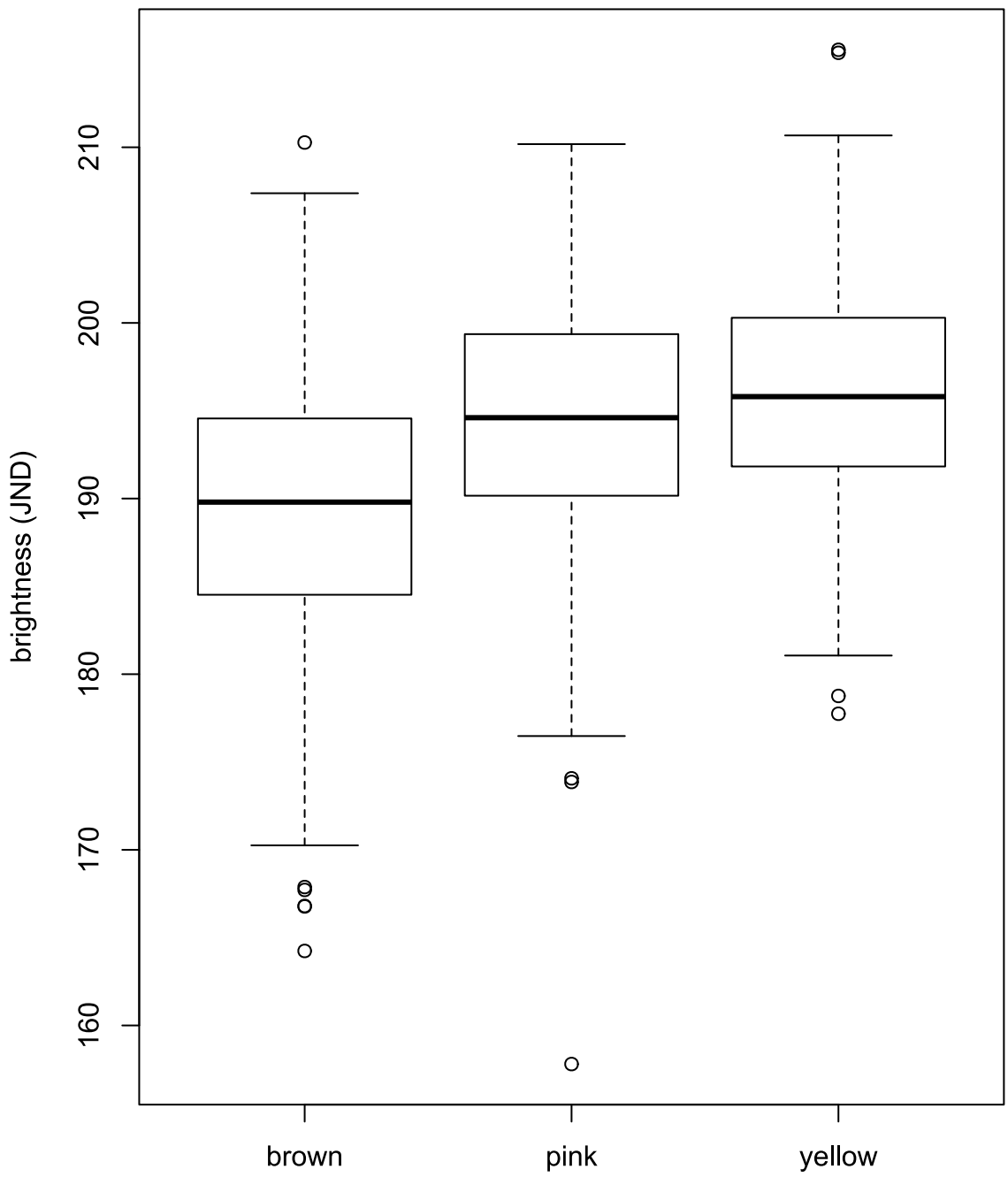

Supplement: Supplementary file 2 — Supplementary Fig. 1 [file 41437_2019_189_MOESM2_ESM.pdf]

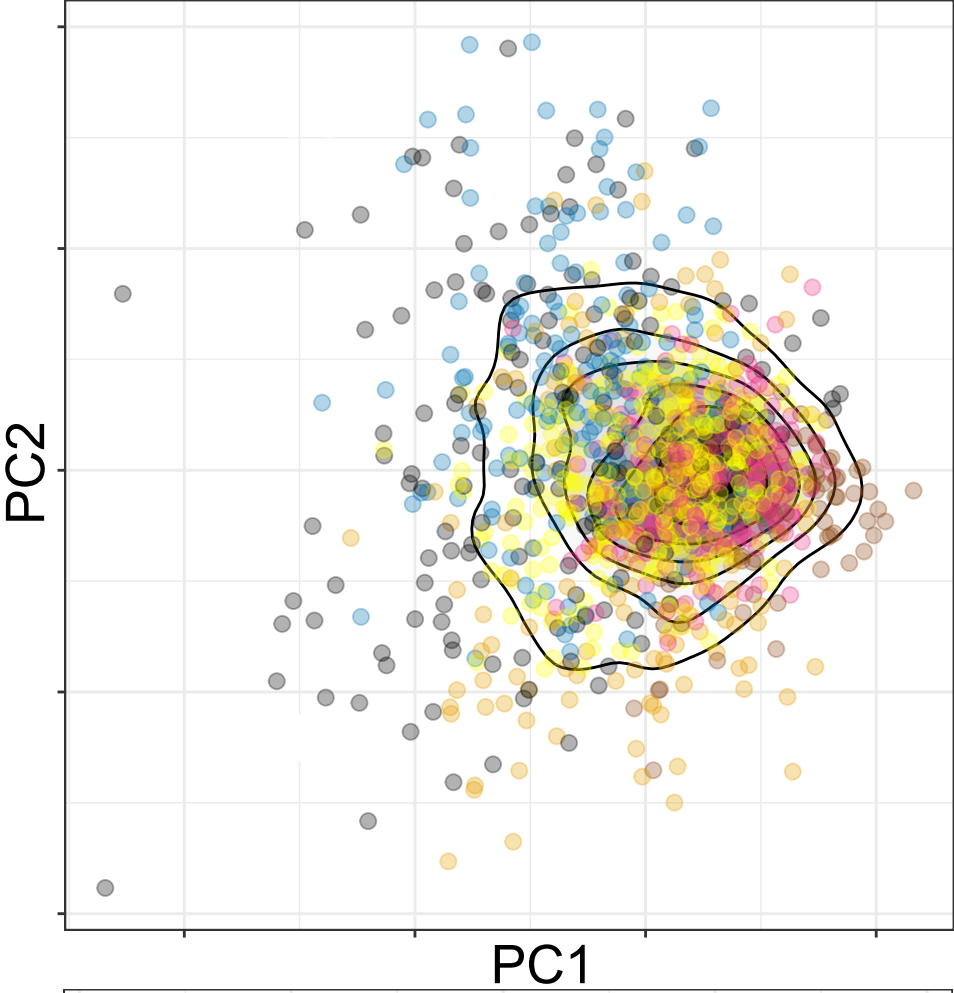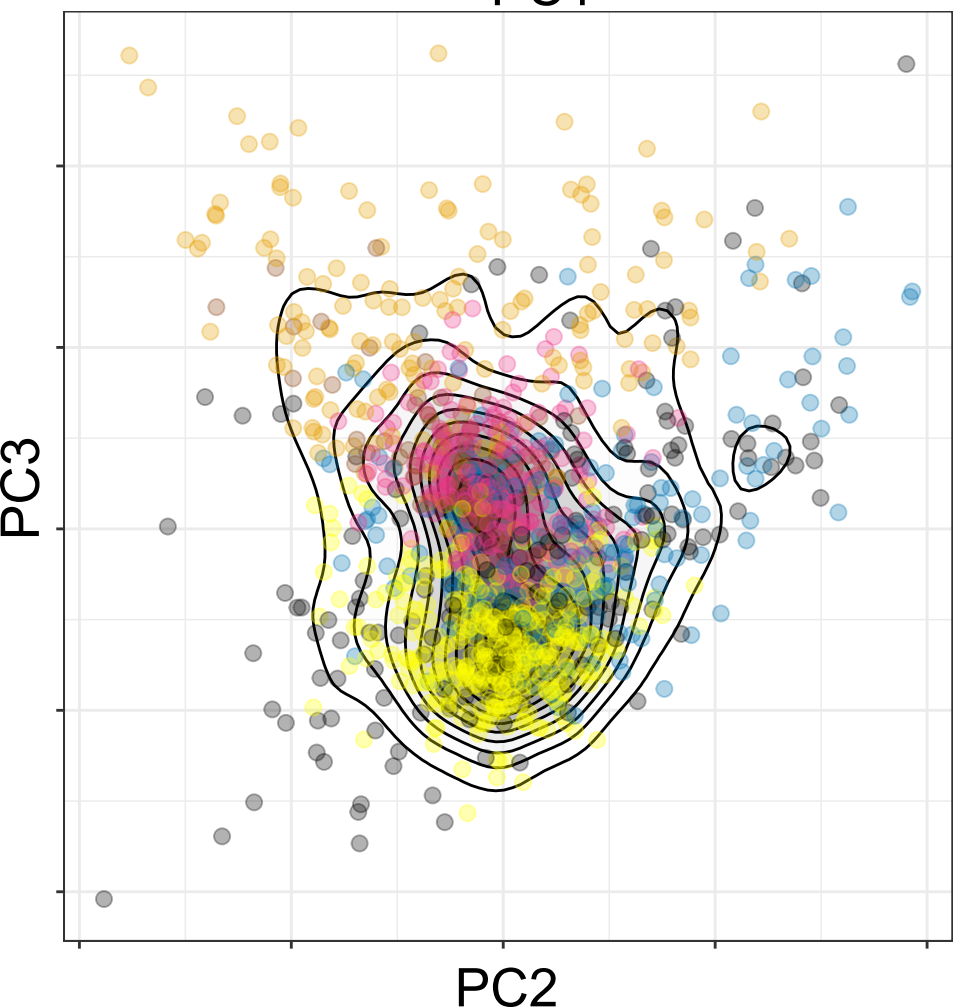

Supplement: Supplementary file 3 — Supplementary Fig. 2 [file 41437_2019_189_MOESM3_ESM.pdf]

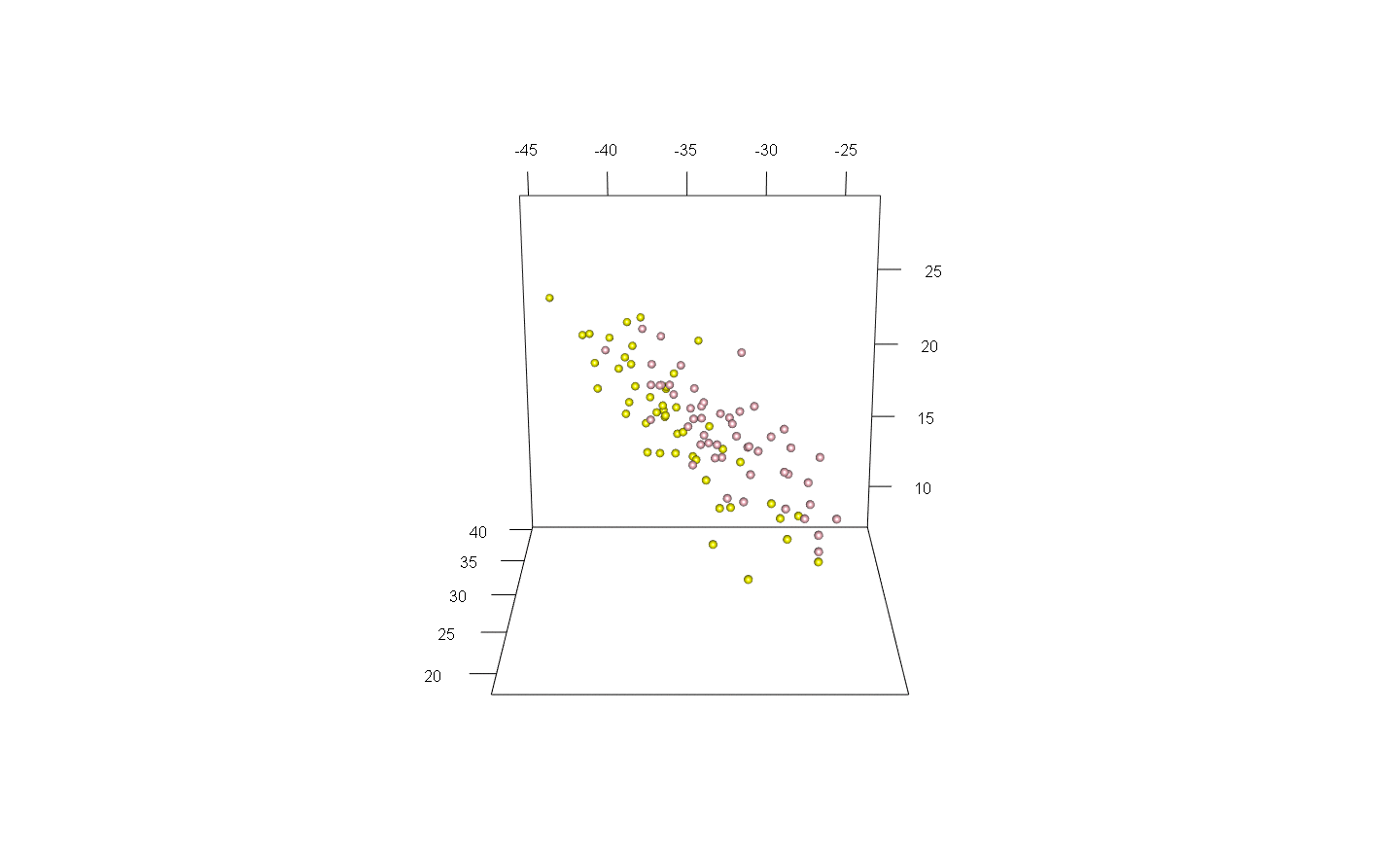

Supplement: Supplementary file 6 — Supplementary movie 1 [file 41437_2019_189_MOESM6_ESM.gif]

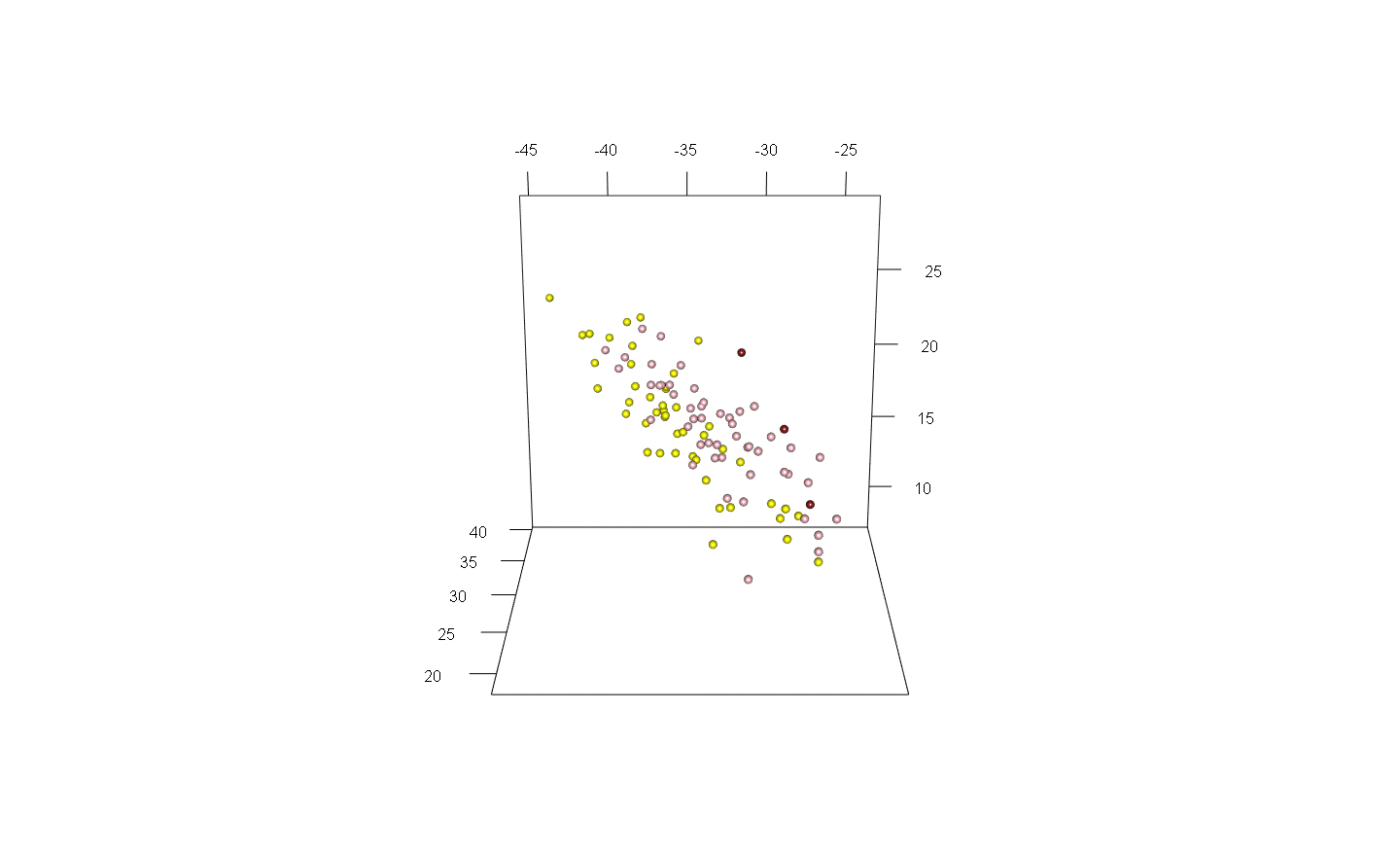

Supplement: Supplementary file 7 — Supplementary movie 2 [file 41437_2019_189_MOESM7_ESM.gif]

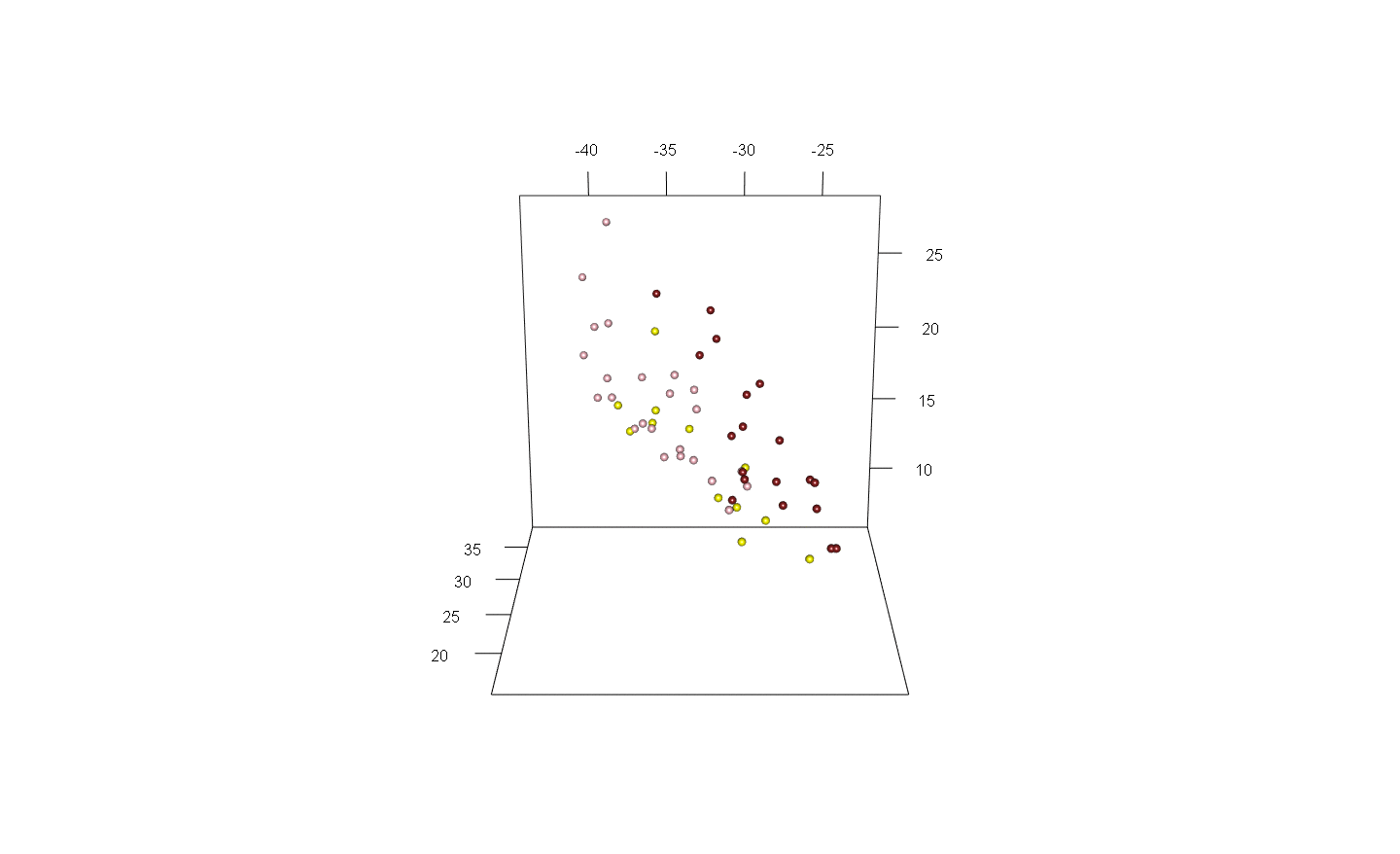

Supplement: Supplementary file 8 — Supplementary movie 3 [file 41437_2019_189_MOESM8_ESM.gif]

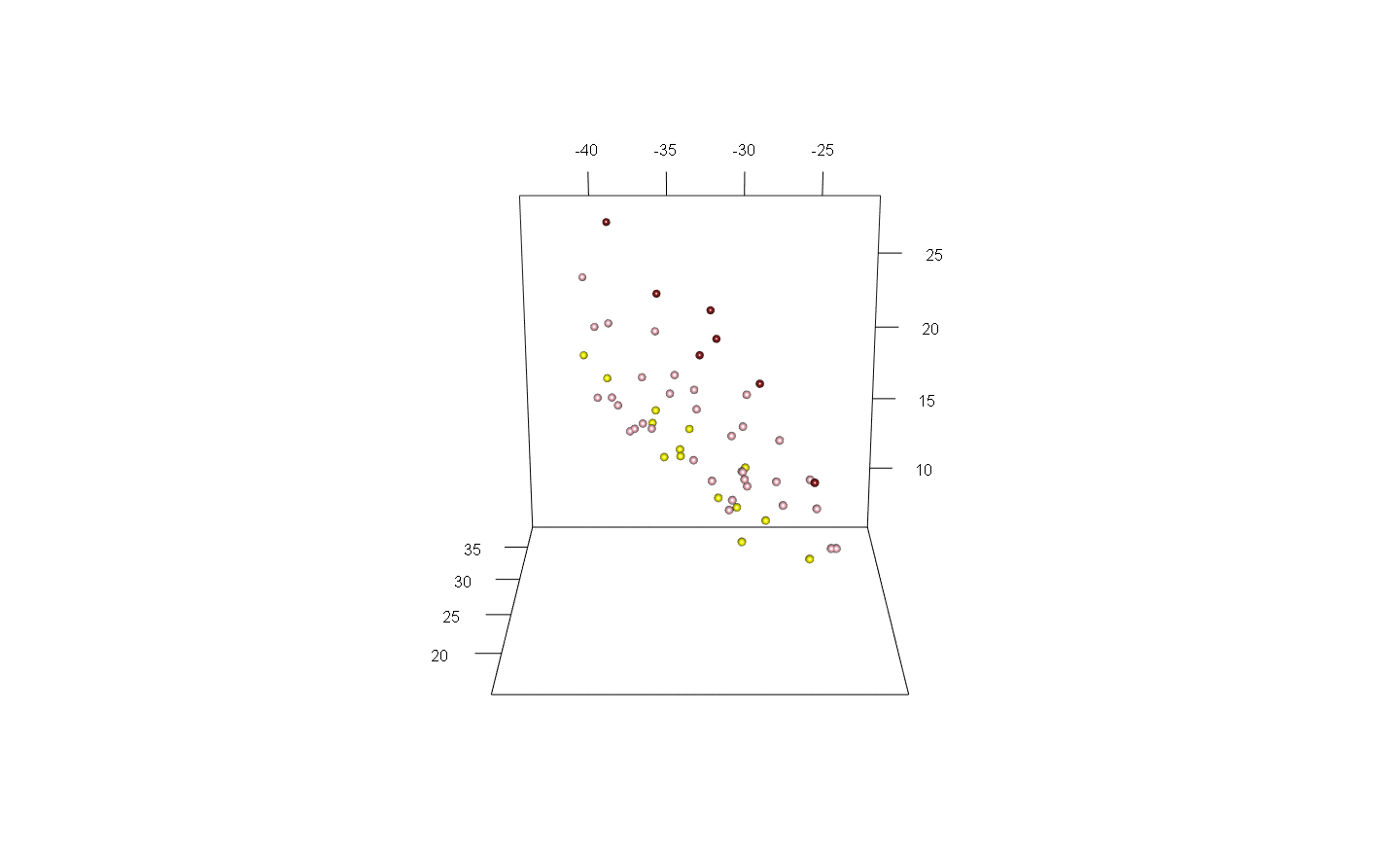

Supplement: Supplementary file 9 — Supplementary movie 4 [file 41437_2019_189_MOESM9_ESM.gif]

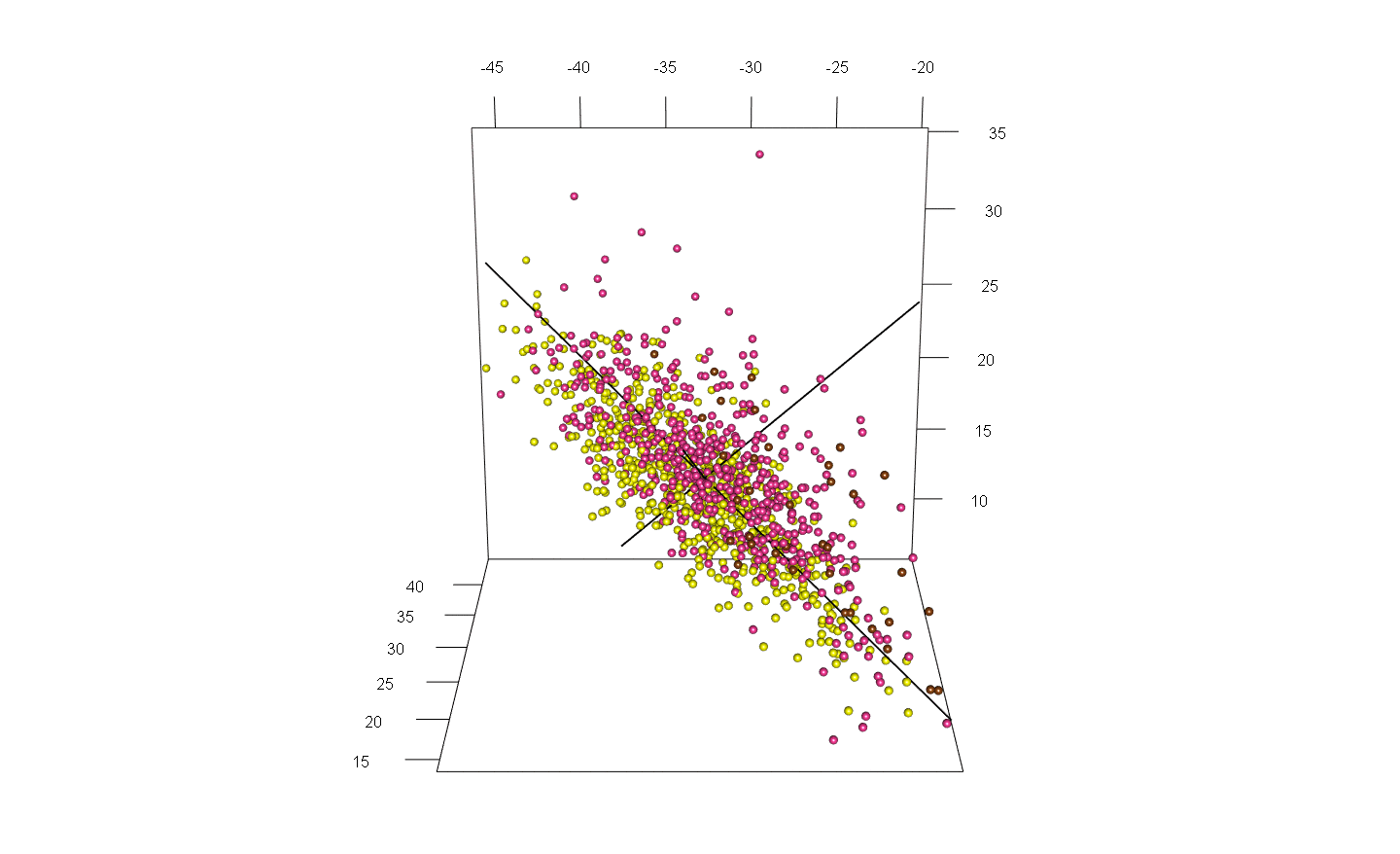

Supplement: Supplementary file 10 — Supplementary movie 5 [file 41437_2019_189_MOESM10_ESM.gif]

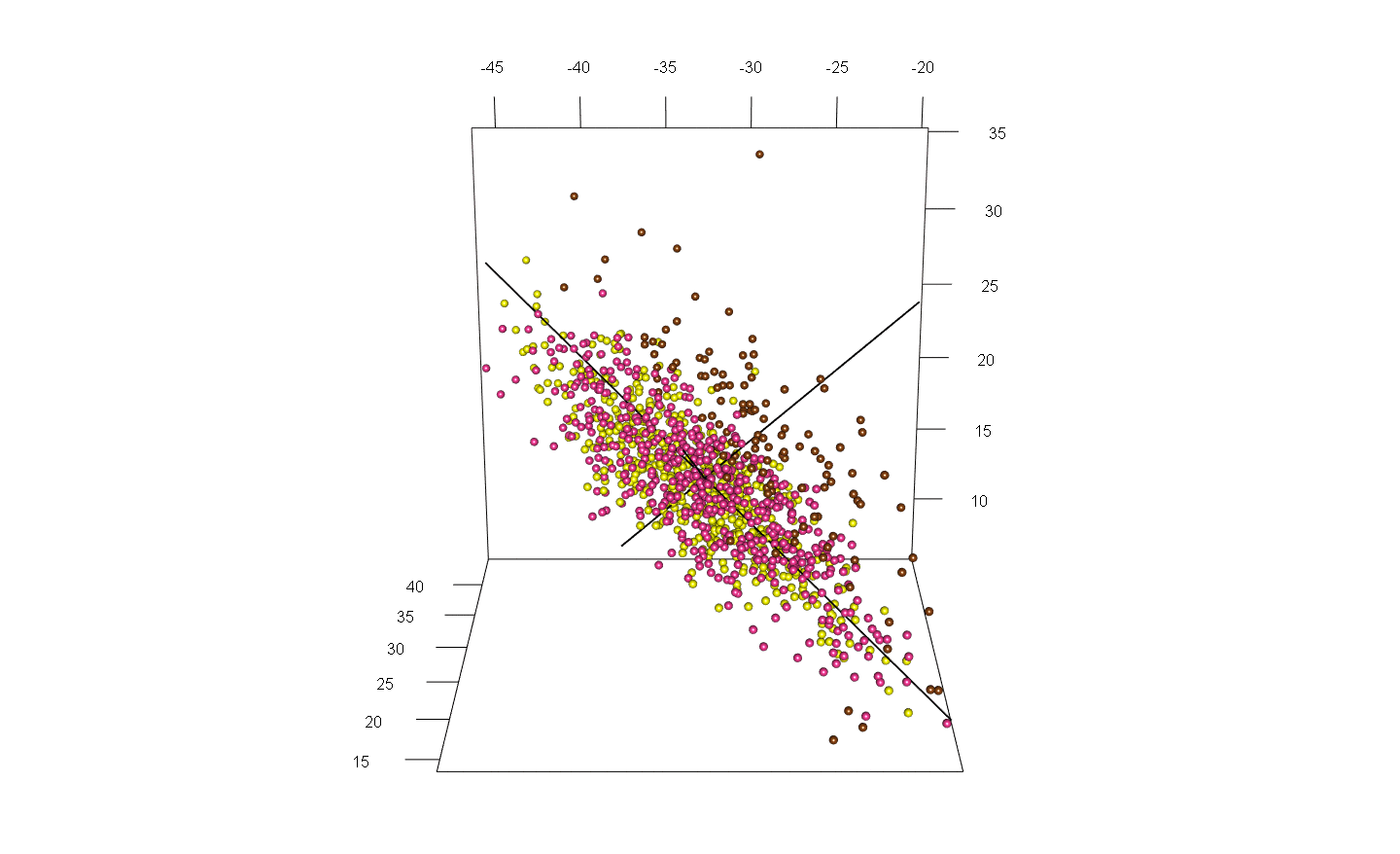

Supplement: Supplementary file 11 — Supplementary movie 6 [file 41437_2019_189_MOESM11_ESM.gif]
